# Intra cavernous aneurysm of internal carotid artery masquerading as a pituitary adenoma: a case report

**DOI:** 10.1186/s13104-018-3329-2

**Published:** 2018-04-10

**Authors:** W. M. U. A. Wijethunga, H. A. Dissanayake, S. Perera, P. Katulanda

**Affiliations:** 1 Diabetes Research Unit, Department of Clinical Medicine, Faculty of Medicine, University of Colombo, No 25, Kynsey Road, Colombo 8, Sri Lanka; 2 Neurosurgery Unit, The Central Hospital, Colombo, Sri Lanka; 3 Clinical Medicine, Faculty of Medicine, University of Colombo, Colombo, Sri Lanka

**Keywords:** Cavernous aneurysm, Pituitary tumour, Carotid aneurysm, Pituitary imaging

## Abstract

**Background:**

Pituitary dysfunction in adults are often associated with tumors of the gland and manifests with mass effects and hypopituitarism. MRI of pituitary region often provides confirmation of the diagnosis and assists in planning neurosurgery.

**Case presentation:**

A 69 years old female evaluated for chronic headache was found to have a supra-sellar mass lesion that mimicked a pituitary tumor, with biochemical evidence of hypopituitarism. Cerebral angiogram confirmed the diagnosis of an aneurysm of the intracavernous internal carotid artery. She was successfully treated with coil embolization of the aneurysm and achieved resolution of symptoms and return of biochemistries to normal.

**Conclusion:**

Carotid aneurysm can mimc pituitary tumours clinically and radiologically on MRI scan. This rare possibility should be considered in evaluating supra-sellar masses to avoid catastrophic consequences.

## Background

Pituitary tumours are the commonest type of intracranial tumours [1]. Their prevalence is reported to be in the range of 5–20% according to autopsy studies [2] and this closely matches the incidence detected in magnetic resonance imaging of otherwise healthy individuals [3].

In contrast, intracranial aneurysms are rare and their incidence range from 0.4 to 3.6% according to autopsy studies and from 3.7 to 6.0% according to studies involving patients undergoing cerebral angiography [4]. Intracranial aneurysms have found to coexist with pituitary adenomas in 2.3–6.9% of patients [5, 6], a rate that is arguably greater than that of the general population. Intracranial aneurysms may rarely present with pseudotumoral syndromes [7], the clinical manifestations determined by the anatomical location of the mass. At times, differentiation between a pituitary neoplasm and a cavernous aneurysm can be challenging [8]. We report a case of intracavernous aneurysm that clinically and radiologically simulated a pituitary neoplasm.

## Case presentation

A 69 years old female presented with gradual onset slowly progressive intermittent diffuse headache of moderate to severe intensity for 3 years. No diurnal pattern of symptoms or associated vomiting or visual disturbance was noted. She was clinically euthyroid and denied galactorrhea, postural dizziness, limb weakness or numbness, convulsions or sphincter dysfunction. Her past history was unremarkable.

General physical examination was unremarkable with normal vital parameters (temperature 37.4 °C, heart rate 76 per min, blood pressure 130/70 mmHg without a postural drop, respiratory rate 16 per min), normal visual fields on confrontation method and had no papilledema or other focal neurological deficits.

Investigations revealed an abnormal thyroid profile suggestive of secondary hypothyroidism [TSH 1.2 mIU/L (0.4–4.0), free T4 0.6 ng/dL (0.9–1.7) and free T3 3.5 pmol/L (3.5–6.5)]. Further investigation showed low cortisol levels (4.03 µg/dL) and markedly raised prolactin level of 1634 mU/L (< 400 mU/L). Thyroxine (75 mcg daily) and hydrocortisone (10 mg mane, 10 mg noon, 5 mg vesper) replacement therapy was commenced and titrated with regular monitoring of free T4 and cortisol levels.

**Fig. 1:**
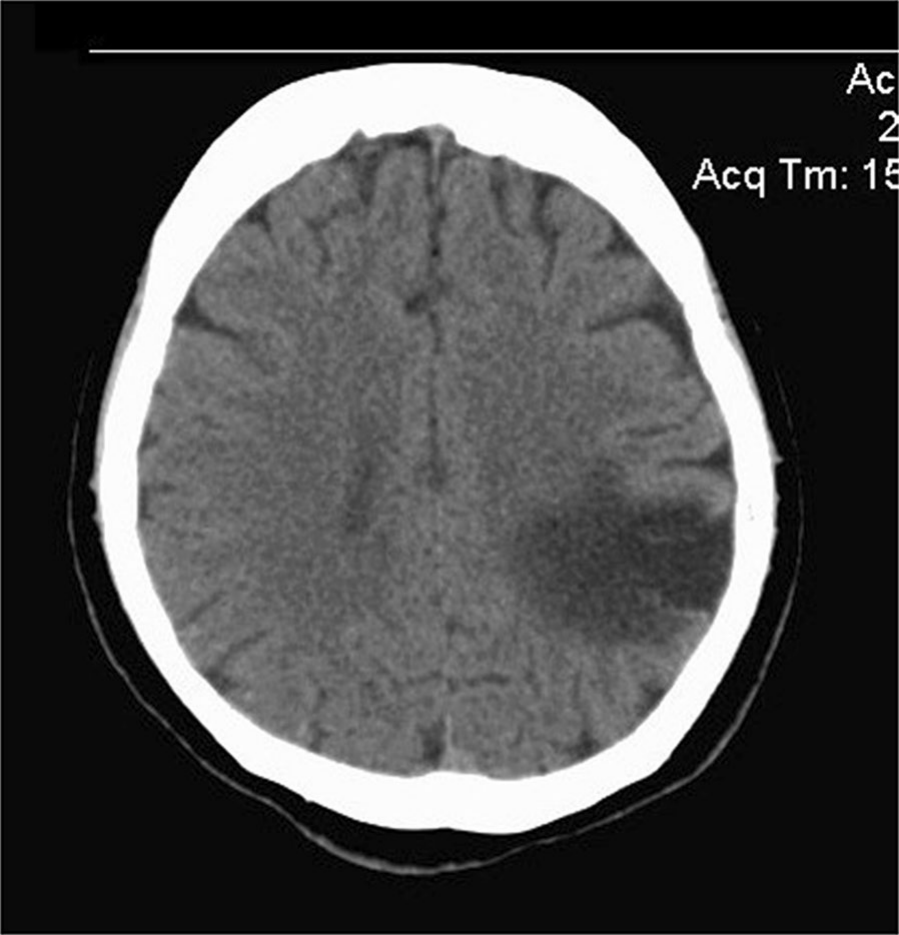
left parietal region infarct seen in non-contrast computerized tomography of brain

**Fig. 2:**
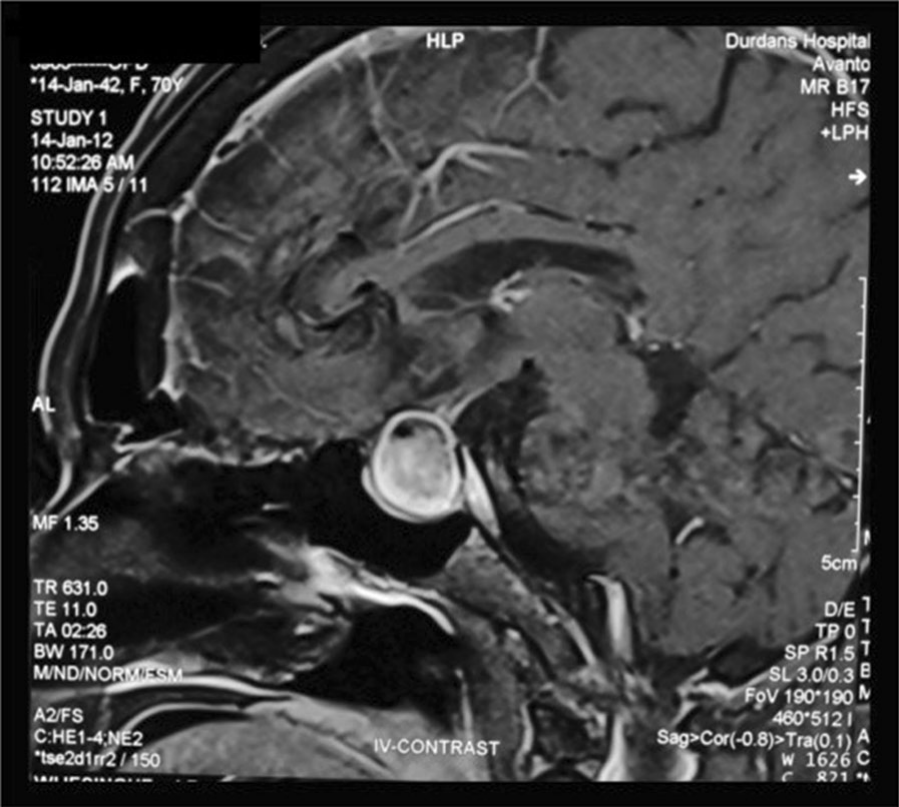
Suprasellar region mass seen in magnetic resonance imaging

Visual fields assessment by perimetry showed a right homonymous inferior quadrantanopia. Non contrast CT scan of the brain showed an old cerebral infarct in the left parietal lobe (Fig. 1). An MRI scan of the head was performed and a mass lesion in the sellar region was found (Fig. 2). The mass lesion was reported as a pituitary macroadenoma and the old cerebral infarct was thought to be unrelated. Patient was referred for neurosurgery after commencing thyroxine and cortisol hormone replacement therapy.

**Fig. 3:**
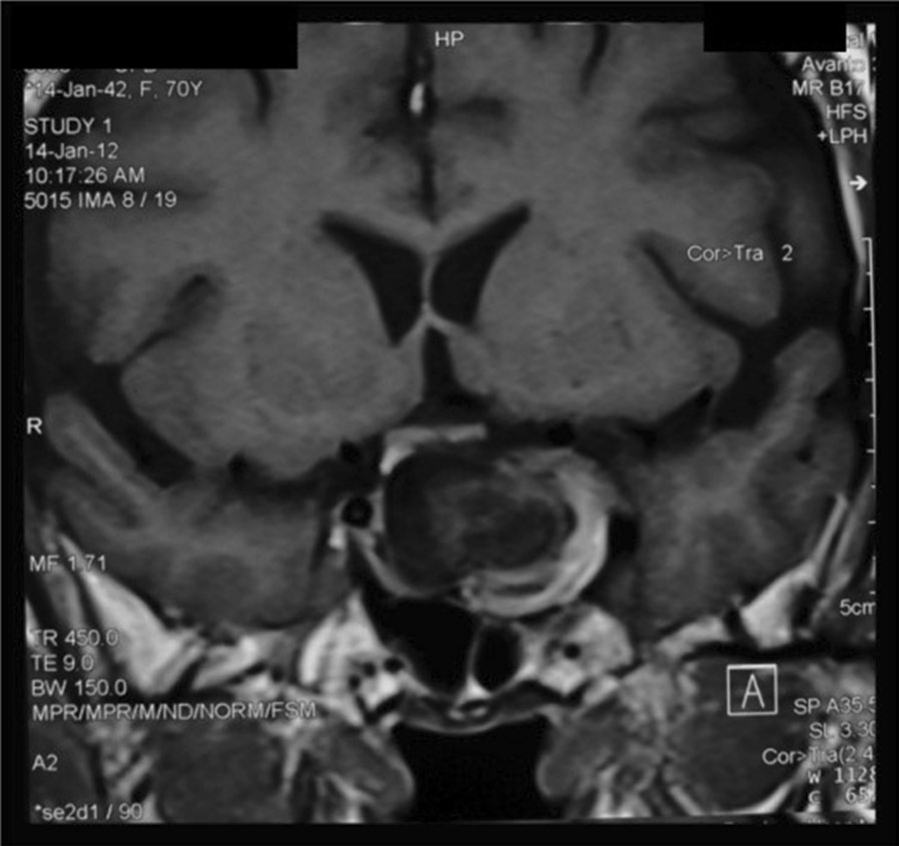
Multi-layered halo like appearance on a coronal section of magnetic resonance imaging

**Fig. 4:**
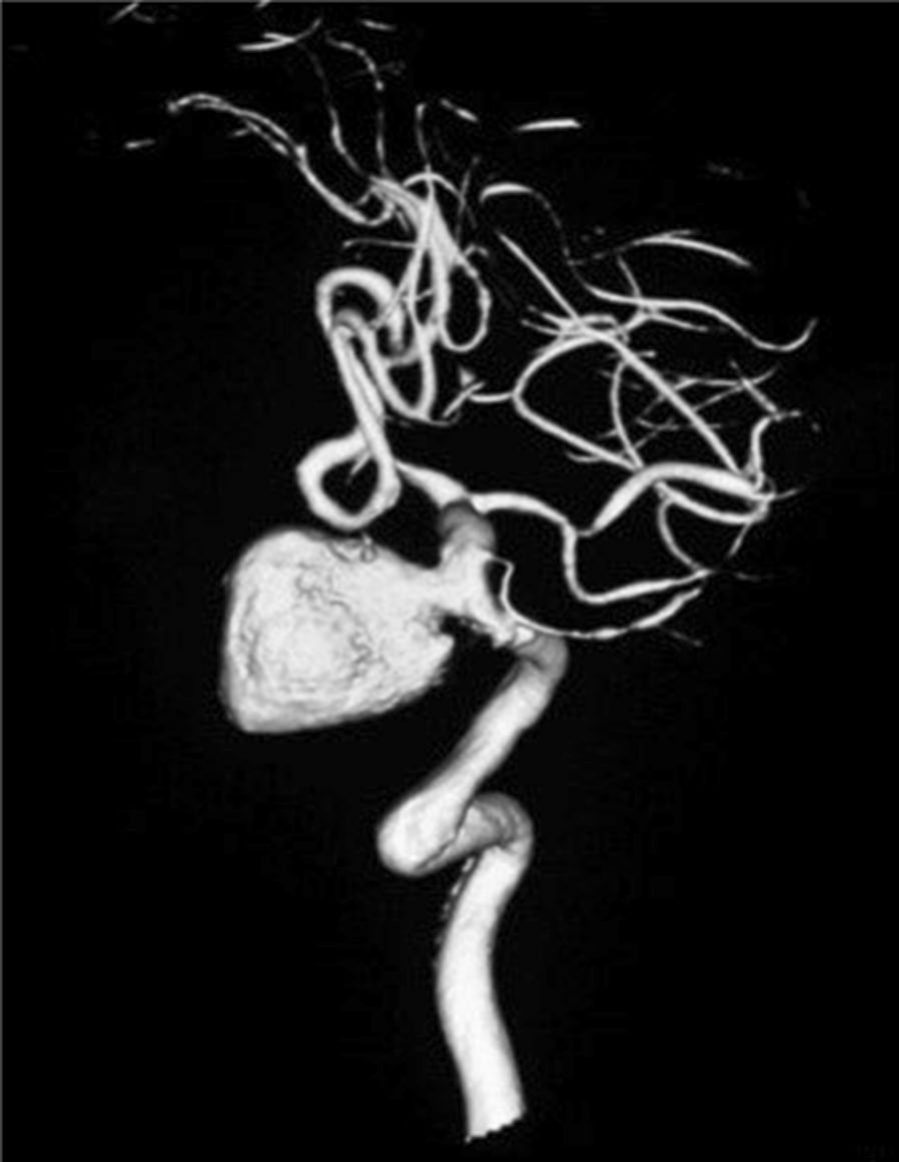
Saccular aneurysm arising from intra-cavernous segment of left internal carotid artery seen in CT angiogram

However on re-evaluation by the neurosurgeon, doubt was cast on the diagnosis of a ‘pituitary tumor’ due to the multilayered and ‘halo’ like appearance of the lesion on MRI (Fig. 3). A CT angiogram was performed due to the unusual appearance of the tumour. A large aneurysm measuring 19.9 mm × 21.5 mm with a neck of 5 mm was discovered arising from the cavernous portion of the left internal carotid artery (Fig. 4).

Thus a potentially catastrophic event was averted which could have occurred had trans-sphenoidal surgery been attempted for a pituitary tumor. It is also noteworthy that old infarct could also have been an embolic consequence of the aneurysm.

**Fig. 5:**
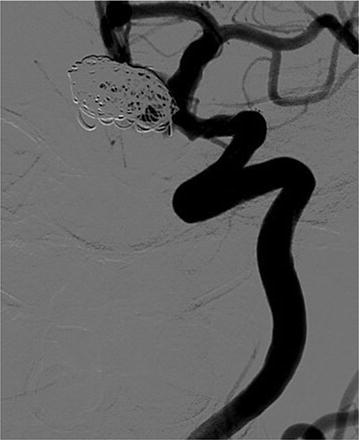
Angiographic appearance after endovascular coiling of the carotid aneurysm

The patient eventually underwent endosaccular coiling (Fig. 5) and stenting without any complications. Her symptoms resolved and made and uncomplicated recovery. However, her visual filed defect did not improve, which would probably have been a result of previous parietal infarct. During a follow up visit at 6 months she remained symptom free but required thyroxine and hydrocortisone replacement.

## Discussion and conclusions

Common presentations of intracranial aneurysms are subarachnoid haemorrhage, cerebral ischaemia or pseudotumoral syndromes [7].

However, intracavernous aneurysms are well known to present with ophthalmoplegia due to compression of third, fourth and sixth cranial nerves as they traverse the cavernous sinus, as well as visual field defects due to chiasmal compression [9, 10]. According to a study in Canada [11] involving 57 patients with giant cavernous carotid aneurysms (> 25 mm), the most common presenting symptoms were diplopia (89%), retro-orbital pain (61%), headache (19%), diminished or blurred vision (14%), and photophobia (4%). While 93% of these patients had had partial or complete ophthalmoplegia trigeminal nerve involvement had been found in 37% of patients. Other clinical signs included ptosis, decreased visual acuity, proptosis, and visual field defects.

Rarely, an intracavernous aneurysm can present as hypothalamo-pituitary axis dysfunction when it extends into the sellar region [8, 12]. Such aneurysms extending into sellar region account for 1–2% of all intracranial aneurysms [13]. However hypopituitarism is very rare as the presenting feature of such tumours and evidence in medical literature is limited to case reports [13]. Review of literature on over 4000 patients with hypopituitarism over four decades concluded that intrasellar aneurysm was the underlying aetiology only in 0.17% of those cases [13].

Intracavernous sinus aneurysms are thought to have a benign course [14]. However, rare but serious complications include meningeal haemorrhage (1.4%), intracavernous fistula (8%) and rarely endocrinological manifestations [14] which warrant surgical treatment. Neurosurgical approach to an intracavernous aneurysm is deemed hazardous and endovascular therapeutic options remain the method of choice [14].

Differentiation between pituitary adenoma and an aneurysm is vital as these two have different management options and mistakenly attempting trans-sphenoidal resection of an aneurysm could have disastrous consequences. Halo appearance produced by the two dural layers encasing the cavernous portion of carotid artery should raise the suspicion of this diagnosis. Filling defects within the mass represent thrombosis. In contrast, a pituitary tumour would appear isointense to grey matter on T1 and T2 weighted MR imaging and would enhance with gadolinium contrast. Large tumours may show heterogeneity and erosion of clinoid processes. Probable higher risk of having an intracranial aneurysm along with the pituitary tumour should also be borne in mind. Infact several authors have reported on subarachnoid haemorrhage during transphenoidal surgery for pituitary adenomas, due to accidental damage to previously undetected intracranial aneurysms [15–17]. Some authors recommend routine preoperative carotid artery angiography in all patients with pituitary macroadenomas before trans-sphenoidal surgery [18].

In summary, this case report illustrates the rare possibility of misidentifying an intracavernous carotid aneurysm as a pituitary tumour. Therefore we emphasize the need of very careful evaluation of the MRI scan before proceeding to pituitary surgery, particularly observing for indirect evidence of an aneurysm such as multi layered halo like appearance, presence of intralesional filling defects and whenever a doubt exists, to perform angiogram to confirm the presence of an aneurysm.

## Abbreviations

MRI: magnetic resonance imaging
TSH: thyroid stimulating hormone
